# Mature or Emerging? The Impact of Treatment-Related Internet Health Information Seeking on Patients’ Trust in Physicians

**DOI:** 10.3390/ijerph15091855

**Published:** 2018-08-28

**Authors:** Runtong Zhang, Xinyi Lu, Wen Wu, Xiaopu Shang, Manlu Liu

**Affiliations:** 1 School of Economics and Management, Beijing Jiaotong University, No. 3 Shangyuancun, Haidian District, Beijing 100044, China; rtzhang@bjtu.edu.cn (R.Z.); xinyilu@bjtu.edu.cn (X.L.); 2 Rochester Institute of Technology, Saunders College of Business, 107 Lomb Memorial Drive, Rochester, NY 14623-5608, USA; manluliu@saunders.rit.edu

**Keywords:** Internet health information seeking, treatments, perceived Internet health information quality, psychological safety, trust in physicians

## Abstract

Years of clinical trials have proven the maturity and safety of certain treatments, however, some of these mature treatments may not be highly effective. Several treatments have emerged through technological innovations, but their long-term safety, efficacy, and adverse effects remain unknown. At present, many patients seek information related to their treatments on the Internet, which may impact their attitudes towards different treatments and their trust in physicians. In this study, a research model was developed to examine how patients’ trust in their physicians is influenced by related online information on mature or emerging treatments. The hypotheses were tested using confirmatory factor analysis (CFA) and structural equation modelling (SEM). A total of 336 valid responses were collected through an online survey. Mature treatments related health information was found to significantly improve patients’ trust. Thus, physicians should pay more attention to mature treatments, and encourage their patients to seek related information online. Moreover, the quality of online information should be developed further to increase patients’ satisfaction. Physicians should also consider their patients’ psychological safety in communication with patients to strengthen their trust.

## 1. Introduction

Treatments can be divided into two categories according to the development conditions: (1) mature treatments, which have been proven safe through years of clinical trials, and (2) emerging treatments, which have emerged in recent years; some emerging treatments are found to be more effective than mature ones, and some are for diseases whose corresponding treatments have not yet been found [1,2,3]. Patients’ demands for health information increase as their health literacy improves [4]. The Internet increases the likelihood of patients seeking treatment-related information online, which may influence their attitudes towards mature or emerging treatments and affect their relationships with their physicians [5]. People are likely to seek health information on the Internet before or after visiting physicians [6,7]. Therefore, they can obtain additional information and knowledge regarding healthcare and their illnesses in addition to those provided by their physicians [8]. However, a previous survey found that patients who are dissatisfied with and who distrust their physicians are more likely to seek health information online [5], and physicians are concerned that health information obtained outside of hospitals may have adverse effects on their relationships with patients [9]. Specifically, patients may choose to trust fragmented information and unprofessional medical knowledge obtained online, but may distrust physicians’ advice and make inappropriate self-diagnoses [4]. Given that the long-term safety, efficacy, and adverse effects of emerging treatments must be examined further [10], physicians may rarely recommend emerging treatments or provide related information to patients. However, some patients may prefer emerging treatments, and they may question their physicians.

Self-management activities are important in ongoing treatments, particularly in cases of chronic diseases [11], and following the advice of physicians is an important premise of self-management. Moreover, only if patients adhere to physicians’ advice can treatment regimens play a role [12]. In physician-patient relationship, patients’ trust in physicians is an important element that positively affects communication, cooperation, and concordance between physicians and patients [12,13]; it also promotes patient compliance with physicians’ advice. Internet health information seeking provides benefits to the health literacy and medication adherence [14]. Thus, the impact of this activity on patients’ trust in their physicians must be determined. Tustin [15] proposed that Internet health information seeking may influence patient compliance with treatments. Laugesen et al. [12] examined the significant impact of Internet health information on patient compliance, but they did not consider patients’ trust in physicians. Lu et al. [16] identified the effects of Internet health information (perceived quality and source) on cognition- and affect-based trust. Krot [17] discussed the relationship between Internet health information seeking and patients’ trust in physicians. However, these researchers did not take the information content into account. Maloney et al. [18] found that patients are more likely to discuss health information related to therapies and treatments with their physicians.

Therefore, this study uses the psychological perspective to examine the impact of treatment-related Internet health information seeking on patients’ trust in physicians. Researchers have begun focusing on behavioural psychology in recent years. However, studies on the relationship between health information and patients’ trust in their physicians remain lacking, particularly in the field of psychology. The present study focuses on patients’ psychological safety. It applies social information processing theory and social exchange theory to patient-physician relationship in the context of Internet health information to determine the impact of patients seeking mature and emerging treatments related Internet health information on patients’ trust in physicians. This study offers theoretical contributions to psychological studies on patient behaviour and physician-patient communication. In addition, it distinguishes between mature and emerging treatments related health information seeking in terms of their impacts on patients’ trust in physicians. Accordingly, this study provides a reference for further study on the relationship between Internet health information and patients’ trust in physicians.

## 2. Research Model and Hypotheses

As a type of human behaviour, information behaviour is associated with information resources and channels. It comprises active or passive information seeking behaviour, information use behaviour, face-to-face communication behaviour, and passive receiving information behaviour. Information seeking behaviour is a purposeful activity that includes information retrieval and browsing; it regards specific requirements as targets [19]. Patients’ demands for health information can be significantly met through health information seeking behaviour [12]. Previous studies have summarised health information into two categories [20,21,22]: (1) healthcare information, which is related to patients’ actual medical issue or illness; and (2) health lifestyle information, which is used to maintain a healthy lifestyle, prevent diseases and manage oneself to improve chronic conditions. As a primary source, the Internet provides convenience to patients in seeking health information [23]. Numerous health information portals have been established by multiple institutions, such as governments, medical institutions and business corporations, to meet the growing demands for health information [24]. At present, patients can obtain health information from different online portals, such as Google, Bing, Yahoo, and many other health-related websites [7]. In addition, online health communities also provide a platform where patients can obtain health information and emotional support from other patients or physicians [25]. These communities allow patients to share their health experiences with others [26]. Such information is beneficial to expand patients’ health-related knowledge and enable them to make lifestyle adjustments [11,27]. Online health information seeking has a positive effect on improving patients’ abilities to solve health-related problems [28]. In some cases, patients with stigmatised illnesses [29] are unwilling to visit physicians. Thus, they prefer to seek relevant health information and then diagnose themselves. In other cases, patients can cure some simple illnesses themselves by using online health information. In addition, patients are occasionally dissatisfied with the treatments provided by their physicians; they may distrust their physicians’ professionalism in conducting treatments, which causes them to seek relevant health information online to verify their physicians’ advice [5,15]. In some instances, patients are likely to discuss Internet health information with their physicians.

The Internet can lead to confusion and uncertainty with regard to health information quality; it also has negative effects on patients, for example, they may obtain health information that mismatches their actual demands [18,30]. Therefore, patients who seek health information online must judge the quality of the content and the sources of the information they consume. In addition, patients have different satisfaction degrees with health information based on their cognitions. Therefore, the perceived quality of and satisfaction with Internet health information must be examined. The association between Internet health information seeking and physician-patient relationship has been the subject of controversial opinions. Some studies have showed that the online information seeking behaviour of patients adversely affects physician-patient relationship. Bell et al. [5] claimed that physicians believe that patients may question their professions, and thus engage in inappropriate self-diagnosis. By contrast, other previous studies have showed that Internet health information seeking has a positive effect on physician-patient relationship [12], and information quality and source exert considerable influences on patients’ cognition- and affect-based trust in physicians [16]. Such conflicting results suggest that other factors must be considered in understanding this relationship. The present study fills in this research gap by identifying how patients’ seeking mature and emerging treatments related online information affects their trust in physicians from the perspective of psychology.

**Figure 1:**
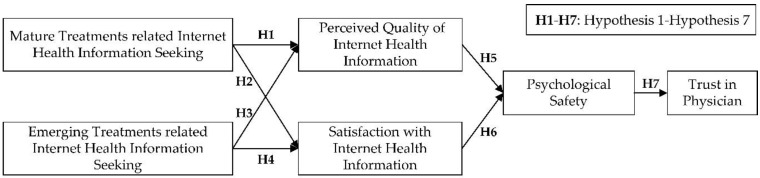
Research Model.

As shown in Figure 1, the research model was developed with six variables and seven hypotheses. The independent variables are mature and emerging treatments related to Internet health information seeking, and the dependent variable is trust in physicians. The mediators include perceived quality of Internet health information, satisfaction with Internet health information, and psychological safety.

Social information processing theory [31] states that individuals seek information from their social environment when the current information is insufficient. Such information may influence their attitudes, beliefs, and opinions [32]. Consequently, it may also influence their behavioural options [33]. In the context of healthcare, patients would like to obtain health information from other sources in addition to their physicians. Different personal backgrounds, characters, and experiences have resulted in various preferences with regard to health information. Regulatory focus theory proposed by Higgins [34] states that individuals are motivated by promotion focus and prevention focus. To specify, individuals with promotion focus pay attention to advancement, growth, and accomplishment. They tend to make progress and approach the desired end-state; they are risk-loving and are more likely to make risky decisions to achieve high success. By contrast, individuals with prevention focus pay attention to security, safety, and responsibility. They prefer to be prudent and precautionary so as to avoid mismatches to the desired end-state; they are risk-averse and are more likely to make repetitive choices or follow routines to avoid risks. This study applies regulatory focus theory to analyze the preferences of patients seeking treatment-related Internet health information. When choosing between mature and emerging treatments, patients with prevention focus prefer low-risk mature treatments and related health information because the uncertainty of technical effectiveness makes them be hesitant to adopt a new technology [35]. In general, this type of health information can be easily and widely obtained via the Internet because it has existed and has been repeatedly verified for many years. Thus, users can compare health information on the same treatments from different sources. Internet users can also identify erroneous information. In addition, Internet health information about mature treatments has relatively high quality and credibility. Thus, patients may perceive high-quality health information. Moreover, risk-averse patients are highly circumspect, such that they think carefully before selecting usable and reliable sources. Consequently, these patients perceive full usefulness and are satisfied with this health information from the Internet. Therefore, we derive the following hypotheses:

**Hypothesis** **H1.** *Mature treatments related Internet health information seeking (MTIHIS) has a positive impact on patients’ perceived quality of Internet health information (PQIHI).*

**Hypothesis** **H2.** *Mature treatments related Internet health information seeking (MTIHIS) has a positive impact on patients’ satisfaction with Internet health information (SIHI).*

By contrast, some patients are risk lovers. They prefer successful results and achieve personal development and self-realisation [34], and thus, they are likely to select challenging treatments and adopt new technology and emerging information [35]. For example, physicians will suggest two treatment options: (1) a mature treatment with a high success rate, but it cannot cure the disease completely; and (2) a new treatment that can be highly successful but involves high risk. Under these conditions, risk-loving patients tend to prefer the risky treatment to pursue high efficiency. Technological innovations have resulted in various risky treatments. Although some of these treatments can be searched online, related health information is generally difficult to find because of the “emerging” feature. Moreover, the quality of available information cannot be guaranteed. Physicians may also lack knowledge of these emerging treatments because of their novelty. Therefore, risk-loving patients prefer to seek related health information on the Internet. However, online health information may have low quality, which may decrease satisfaction. This situation leads to the following hypotheses:

**Hypothesis** **H3.** *Emerging treatments related Internet health information seeking (ETIHIS) has a negative impact on patients’ perceived quality of Internet health information (PQIHI).*

**Hypothesis** **H4.** *Emerging treatments related Internet health information seeking (ETIHIS) has a negative impact on patients’ satisfaction with Internet health information (SIHI).*

Psychological safety plays an important role in cooperative relationships and promotes individuals’ willingness to ask for help, seek feedback, free expression, share information, and exchange knowledge with others without fear of potential negative consequences or interpersonal risks [36,37,38]. Psychological safety has been investigated from the aspects of improving team performance, individuals’ happiness, team cohesiveness, and motivation to share information [39,40]. Patients and physicians form a special cooperative relationship with the target of successfully completing the treatment process. Therefore, psychological safety is essential for patients to establish a secure medical treatment environment that promotes reliance on physicians, which allows patients to disclose the actual state of their illness, exchange information with physicians, and follow the self-management advice of physicians after treatments [41]. Patient safety has six aspects: safety and security of public spaces, safety of medical services, privacy and information security, financial security, psychological safety, and gap in services. Wang et al. [42] found that over half of the patients focus more on psychological safety. In addition, psychological safety is partially relevant to distrust in physician-patient relationship [43]. Unlike patients, however, physicians are likely to concentrate on organizing technical services [42]. Therefore, psychological safety is an important element in examining the impact of Internet health information seeking on patients’ trust in physicians. Patients who adopt health information from their social environments will have different attitudes, beliefs, and opinions [32]. Patients who perceive high quality of health information obtained online and are satisfied with this information may believe that their health information literacy is high; thus, they may assume that they are capable of helping physicians during treatments, which reduces anxiety whilst fostering a positive attitude [31,44]. In such case, patients may be able to overcome psychological barriers and improve their sense of psychological safety [45]. Hence, we propose the following hypotheses:

**Hypothesis** **H5.** *Patients’ perceived quality of Internet health information (PQIHI) has a positive impact on patients’ psychological safety (PS).*

**Hypothesis** **H6.** *Patients’ satisfaction with Internet health information (SIHI) has a positive impact on patients’ psychological safety (PS).*

Trust plays a central role in physician-patient relationship [46,47]. It makes patients feel secure and comfortable; it also motivates them to report their conditions and follow their physicians’ treatment advice [48]. As a type of interpersonal trust, which is defined as ‘the extent to which a person is confident in, and willing to act on the basis of, the words, actions and decisions of another’, patients’ trust is described as patients’ belief in the performance of physicians; it is divided into two aspects: cognition- and affect-based trust [49]. On the one hand, patients are likely to trust physicians whom they perceive to be professional based on their cognitions. Patients follow the advice of these physicians during treatments and self-management after diagnoses. On the other hand, patients rely on their physicians and expose their vulnerabilities if they perceive strong emotional communication that may make them feel secure during treatments [48]. Patients’ trust contributes to the effectiveness of a healthcare system; it plays an important role in diagnosis, treatment, and recovery [50,51]. Lack of trust may destroy physician-patient relationship and shorten its duration [52]. In addition, patients’ trust is important in strengthening physician-patient relationship, improving patients’ compliance with medical diagnoses and treatment regimens, and increasing the utilisation of health services [51,53,54,55]. Trust is a dynamic parameter [56] that can be influenced by several factors, such as physicians’ ability and integrity [57], patients’ mental status, and physician-patient communication. Physician-patient relationship includes an exchange of resources, such as love, status, information, money, goods and services [58,59,60]. Patients require accurate information and suitable treatments, and physicians pursue patients’ satisfaction and society reputation. Therefore, patients’ trust in their physicians always depends on whether patients perceive sincere treatments from their physicians. Patients who perceive high psychological safety are willing to exchange resources with physicians, such as communicating, sharing views, and asking questions on their illness [58,61]. Such exchange of resources is beneficial for medical interaction. Given that patients’ trust is influenced by the communication between physicians and patients, patients with high psychological safety may have increased trust in their physicians [62]. Hence, we propose the following hypothesis:

**Hypothesis** **H7.** *Patients’ psychological safety (PS) has a positive impact on patients’ trust in physicians (TP).*

## 3. Materials and Methods

### 3.1. Instrument Development

We adopted instruments from previous studies to ensure the reliability and validity of scales. Multiple-item scales with a 7-point Likert-type format that ranges from “strongly disagree” to “strongly agree” were used to measure six constructs that cover all the variables, as shown in Table A1 (Appendix A). A 5-item scale developed by Lemire et al. [63] was used to measure mature and emerging treatments related Internet health information seeking. We also adapted a 3-item scale from Chikoko et al. [64] to measure patients’ sense of psychological safety. Patients’ perceived quality of Internet health information is a second-order construct that includes relevance, understandability, adequacy, and usefulness; we measured it using a 16-item scale [12]. Satisfaction with Internet health information was measured using an 11-item scale from Lemire et al. [63], and patients’ trust in their physicians was measured using a 10-item scale adapted from Torbit et al. [65].

### 3.2. Analysis Tool Selection

In consideration of the mediators and complex relationships in the research model, we used structural equation modelling (SEM) to test hypotheses to incorporate the measurement errors and detect effects. In addition, the confirmatory factor analysis (CFA) was performed to examine the discriminant validity of scales and to combine with SEM to improve the research model [66]. Since there were latent variables in the research model, we used IBM SPSS Statistics 22.0 and AMOS 22.0 to analyse data, which can achieve efficient and unbiased analysis.

### 3.3. Data Collection and Respondent Profile

The present study processed the scales using methods adopted from previous studies [67,68] for cross-cultural adaptation and consistency [69]. English scales were translated into Chinese to ensure that the Chinese participants could read and answer the questionnaire. To specify, we recruited three native Chinese speakers who are fluent in speaking English and who are proficient in scientific research to help with the translation. Then, we modified the translated scales to improve understandability, appropriateness, and readability. We invited 10 individuals with different backgrounds to read and answer the scales. Such trial was conducted to determine the clarity, conciseness, and total required time to accomplish the questionnaire. Finally, we ensured the consistency between the original and the Chinese versions of the scales through a back-translation process.

The survey was conducted online in June 2017. Our subjects were Chinese individuals who had received therapies within a month and had experiences in seeking health information online. Therefore, the responses will be representative to analyse and develop the research model that related to Internet health information seeking. With the help of a medical association in China, questionnaires were sent to 486 participants who had the experience of treatment with a month so that they were able to clearly recall the experiences and the feelings. The survey was anonymous to guarantee the respondents’ privacy. Informed consent was also secured for each participant before the survey. We received a total of 375 responses; among which, 336 were valid. The respondents’ demographic information is provided in Table 1. More than half of the subjects were female, highly educated and below 40 years old. We believe that the sample meets the requirements of Internet health information seeking because young, female, and educated individuals are more likely to use the Internet to seek for health information [4,70].

## 4. Results

### 4.1. Data Analysis

This study used CFA and SEM to test the hypotheses. The results using SPSS 22.0 (IBM, Armonk, NY, USA) proved acceptable reliability because each Cronbach’s α of the constructs was above the cut-off value of 0.700 [71], as shown in Table 2. In addition, the Kaiser-Meyer-Olkin (KMO) value was 0.905, which denoted the perfect acceptability of the construct validity (weak: 0.500; medium: 0.600; good: 0.700; very good: 0.800; perfect: 0.900) [72,73,74,75]. The discriminant and convergent validities of the constructs was tested via CFA. Table 3 presents the fit indices of the research model, which exhibit a good fit to the data [76,77,78,79,80]. In addition to construct validity, discriminant and convergent validities should also be evaluated. Table 4 provides the values of the mean, standard deviation (SD), composite reliability (CR) and average variance extract (AVE), and Table 5 shows the correlation between each two constructs. The value of AVE exceeds 0.500 and CR is above 0.700, which indicates an adequate convergent validity; meanwhile, discriminant validity can be acceptable under the circumstance that each construct’s square root of AVE is higher than its correlations with the other constructs [81,82]. Therefore, we can conclude that the discriminant and convergent validities of the scales are acceptable.

### 4.2. Hypotheses Testing

We analysed the effects of several demographic factors on the research model using *t*-test, analysis of variance, and analysis of covariance. The results indicated that age, gender, and education level did not have any significant effect on the constructs, whereas living area had a significant effect on mature and emerging treatments related Internet health information seeking. Patients who lived in urban areas were more likely to seek online health information on mature treatments. In addition, job exerted a significant effect on patients’ Internet health information seeking. Compared with factory workers, students, professionals, private business owners, and commercial service workers were more willing to seek health information regarding emerging treatments on the Internet. Therefore, we can conclude that our sample are representative in terms of the age, gender, and education level. In addition, we added living area and job as control variables into the research model.

**Figure 2:**
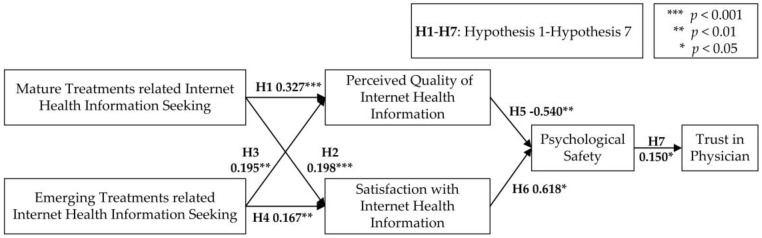
Research Model with Path Coefficients and Significances.

Figure 2 and Table 6 show the path coefficients and their corresponding significances. H1, H2, H6 and H7 were supported, whereas H3, H4 and H5 were not supported because the directions of influences were inconsistent with the original hypotheses. To specify, our results indicated that both mature treatments and emerging treatments related Internet health information seeking had a positive impact on patients’ perceived quality of Internet health information and their satisfaction with such information. Satisfaction with Internet health information had a positive impact on psychological safety. Meanwhile, patients’ perceived quality of Internet health information had a negative impact on their psychological safety. In addition, patients’ psychological safety had a positive impact on their trust in physicians.

## 5. Discussion

This study has several theoretical and practical implications for future research on Internet health information seeking, physician-patient communication and relationship. Firstly, we established a research model for identifying the impact of Internet health information seeking on patients’ trust in their physicians, which was mediated by patients’ perceived quality of information and satisfaction with this information. We clarified how patients’ treatment-related online health information seeking influences their trust during communication with physicians. To specify, mature treatments related Internet health information seeking has a direct, positive impact on patients’ perceived quality of Internet health information and satisfaction with such information, and an indirect impact on patients’ psychological safety and trust in physicians. Patients are more likely to perceive high-quality Internet health information and be satisfied with this information if they seek health information regarding mature treatments on the Internet. In accordance with Bylund et al. [4], high satisfaction with Internet health information promotes the feeling of psychological safety among patients when they communicate with their physicians, thereby improving patients’ trust in physicians. However, the result of the present study shows that patients’ perceived quality of Internet health information has a negative effect on psychological safety, and thus, H5 is not supported. We assumed that the relationship between perceived quality of Internet heath information and psychological safety may be influenced by satisfaction with the information. Therefore, we conducted a follow-up analysis to examine this effect. Then, the perceived quality of Internet health information was found to have a positive effect on psychological safety when the influence of satisfaction with Internet health information was ignored. In addition, further analysis indicated that the perceived quality of Internet health information has a direct, positive effect on satisfaction with Internet health information, and an indirect, positive effect on psychological safety.

Secondly, emerging treatments related Internet health information seeking significantly affects the perceived quality of Internet health information and satisfaction with this information. However, the directions of these effects contradict H3 and H4. Emerging treatments related information seeking has definite positive effects on patients’ attitudes (i.e., perceived quality and satisfaction) towards online health information. To specify, patients who seek health information online frequently have high e-health literacy [7]. They can distinguish among the qualities of different health information and obtain appropriate treatment-related health information on the Internet. Therefore, patients who prefer Internet health information related to emerging treatments are also likely to gain high-quality online health information and be satisfied with this information. In addition, the path coefficients from mature treatments related Internet health information seeking to patients’ perceived quality of information and their satisfaction with such information was higher than the corresponding coefficients from emerging treatments related Internet health information. Thus, mature treatments related Internet health information seeking has a stronger impact than emerging treatments related Internet health information seeking on perceived information quality and satisfaction with such information. These findings suggest that physicians should pay more attention to mature treatments related Internet health information, and encourage patients to do the same to enhance the quality of physician-patient communication and patients’ trust in physicians. In addition, the quality, usability, usefulness, and accessibility of healthcare portals that provide health-related information must be moderated to improve patients’ satisfaction with online health information.

**Figure 3:**
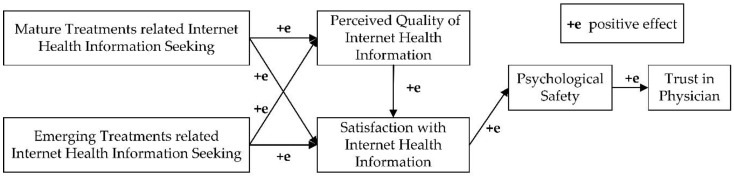
Modified Research Model.

Thirdly, with regard to treatments, patients’ psychological safety has a positive impact on their trust in their physicians, which is one of the premises of communication between patients and physicians. Communication plays a vital role in physician-patient relationship [83]. Gordon et al. [62] found that communication affects trust in physician-patient relationship. Patients with high perceived high psychological safety may feel free to communicate with their physicians, express their viewpoints, share health information and ask for physicians’ suggestions. Therefore, these patients can learn more from physicians through communication [84], which further improves trust in the physician-patient relationship. Moreover, physicians must focus on patients’ psychological states and avoid harming their psychological safety. Physicians should create a safe atmosphere for communication to improve patients’ psychological safety, thereby encouraging patients to freely express themselves, talk about their conditions, cooperate with treatments, and eventually strengthen the quality of diagnoses and treatments. From the preceding discussions, we modified the research model, as shown in Figure 3.

This study has several limitations. Firstly, its mediators are limited to patients’ perceived quality of information and satisfaction with such information. Other mediators may be discussed in future studies. Secondly, we intend to develop the mechanism for identifying how emerging treatments related online health information seeking negatively impact patients’ trust in physicians in future studies, which has not been examined in this study. Thirdly, this study examined the impacts of treatment-related online health information seeking on patients’ trust in their physicians in the context of China. However, the healthcare system and its development are distinctive in China. Thus, this research model should be tested and developed in other countries, and compared with that in China. Fourthly, the sample in this study is relatively young because our subjects are individuals who seek health information on the Internet. However, repeating the study years later when all the age spectrum will be Internet users is interesting. Fifthly, although the sample was consistent with the characteristics of Internet health information seekers, we failed to accurately discuss the representativeness of this study population in terms of the total population of China since we did not consider the feature of Chinese census data. Sixthly, we can ensure that our participants all had received therapies within a month and had experiences in seeking health information online, but we did not collect the data of their treatment frequencies and online health information seeking frequencies and relevant length of time in the questionnaire, so we cannot quantitatively analyse the representativeness of our sample. Seventhly, all the constructs were measured only once, and thus the findings may be accidental. Finally, the relationships were evaluated statistically without external validation, which can be developed for future research.

## 6. Conclusions

This study explores how patients’ treatment-related Internet health information seeking impacts their trust in physicians through the mediation of perceived quality of information, satisfaction with information, and psychological safety. In our research model, mature and emerging treatments related Internet health information have direct, positive impacts on the perceived quality of Internet health information and satisfaction with this information. The perceived quality of Internet health information has a direct, positive impact on satisfaction with information, and an indirect, positive impact on psychological safety. Satisfaction with Internet health information has a direct, positive impact on psychological safety. Psychological safety has a direct, positive impact on patients’ trust in physicians. Moreover, mature treatments related Internet health information seeking plays a stronger role than emerging treatments related Internet health information seeking in improving patients’ perceived quality of information and their satisfaction with such information, and consequently enhancing patients’ trust in physicians. These findings suggest that the following. (1) Physicians should pay increased attention to mature treatments related Internet health information, and encourage patients to do the same. (2) The quality of online health-related information, particularly its usability, usefulness, and accessibility, should be moderated. (3) Physicians are advised to focus on patients’ psychological safety, and to encourage patients to express themselves freely.

## Figures and Tables

**Table 1:** Sample Demographics.

Demographic Characteristics		Number	Percentage
(1) Age	<20	22	6.55%
	21–29	83	24.70%
	30–39	107	31.84%
	40–49	59	17.56%
	50–59	47	13.99%
	60 and above	18	5.36%
(2) Gender	Male	156	46.43%
	Female	180	53.57%
(3) Resident Status	Urban	184	54.76%
	Rural	152	45.24%
(4) Education Level	Junior middle school	31	9.22%
	High school	96	28.57%
	Junior college	68	20.24%
	Bachelor’s degree	127	37.80%
	Master’s degree	9	2.68%
	Doctor’s degree	5	1.49%
(5) Job	Private business owners	28	8.33%
	Factory workers	31	9.23%
	Professional and technical workers	77	22.92%
	Commercial service workers	63	18.75%
	Students	38	11.31%
	Liberal professionals	27	8.04%
	Employees in government offices and public institutions	40	11.90%
	Retirees	22	6.55%
	Farmers	10	2.98%

**Table 2:** Cronbach’s α of Constructs.

Construct ^a^	Cronbach’s α
MTIHIS	0.854
ETIHIS	0.844
PQIHI	0.933
SIHI	0.891
PS	0.867
TP	0.842
Total	0.936

^a^ MTIHIS = Mature Treatments-related Internet Health Information Seeking; ETIHIS = Emerging Treatments-related Internet Health Information Seeking; PQIHI = Perceived Quality of Internet Health Information; SIHI = Satisfaction with Internet Health Information; PS = Psychological Safety; TP = Trust in Physician.

**Table 3:** Fit Indices.

Fit Indices	Values	Threshold for a Good Fit
Pearson’s Chi-square (χ^2^)	1015.968	-
Degrees of freedom (*df*)	893	-
χ^2^/*df*	1.138	<3
Probability level (*p*)	0.000	<0.001
Root mean square error of approximation (RMSEA)	0.020	<0.05
Root mean square residual (RMR)	0.078	<1
Goodness of fit index (GFI)	0.895	≥0.900
Comparative fit index (CFI)	0.986	≥0.900
Normed Fit Index (NFI)	0.895	≥0.900
Relative Fit Index (RFI)	0.862	≥0.900
Incremental Fit Index (IFI)	0.986	≥0.900
Tucker-Lewis Index (TLI)	0.981	≥0.900

**Table 4:** Mean, SD, CR and AVE.

Construct ^a^	Mean	S.D.	CR	AVE	Sqrt AVE
MTIHIS	4.685	0.827	0.878	0.591	0.769
ETIHIS	4.214	0.916	0.874	0.582	0.763
PQIHI	4.503	0.748	0.905	0.426	0.653
SIHI	4.473	0.822	0.847	0.445	0.667
PS	3.638	1.123	0.748	0.599	0.774
TP	4.180	0.826	0.915	0.522	0.723

^a^ MTIHIS = Mature Treatments-related Internet Health Information Seeking; ETIHIS = Emerging Treatments-related Internet Health Information Seeking; PQIHI = Perceived Quality of Internet Health Information; SIHI = Satisfaction with Internet Health Information; PS = Psychological Safety; TP = Trust in Physician.

**Table 5:** Correlations of Latent Variables.

Construct ^a^	1	2	3	4	5	6
MTIHIS	0.769					
ETIHIS	0.291	0.763				
PQIHI	0.362	0.286	0.653			
SIHI	0.305	0.295	0.749	0.667		
PS	−0.149	−0.019	−0.119	−0.001	0.774	
TP	0.361	0.212	0.495	0.525	0.115	0.723

^a^ MTIHIS = Mature Treatments-related Internet Health Information Seeking; ETIHIS = Emerging Treatments-related Internet Health Information Seeking; PQIHI = Perceived Quality of Internet Health Information; SIHI = Satisfaction with Internet Health Information; PS = Psychological Safety; TP = Trust in Physician.

**Table 6:** Hypotheses Testing Results.

Hypothesis	Path Coefficient	*p* ^a^
**H1**: Mature treatments related Internet health information seeking (MTIHIS) has a positive impact on patients’ perceived quality of Internet health information (PQIHI).	0.327	***
**H2**: Mature treatments related Internet health information seeking (MTIHIS) has a positive impact on patients’ satisfaction with Internet health information (SIHI).	0.198	***
**H3**: Emerging treatments related Internet health information seeking (ETIHIS) has a negative impact on patients’ perceived quality of Internet health information (PQIHI).	0.195	0.002 **
**H4**: Emerging treatments related Internet health information seeking (ETIHIS) has a negative impact on patients’ satisfaction with Internet health information (SIHI).	0.167	0.001 **
**H5**: Patients’ perceived quality of Internet health information (PQIHI) has a positive impact on patients’ psychological safety (PS).	−0.540	0.002 **
**H6**: Patients’ satisfaction with Internet health information (SIHI) has a positive impact on patients’ psychological safety (PS).	0.618	0.018 *
**H7**: Patients’ psychological safety (PS) has a positive impact on patients’ trust in physicians (TP).	0.150	0.045 *

^a^ *** *p* < 0.001, ** *p* < 0.01, * *p* < 0.05.

## Appendix A

**Table A1:** Measurement Instrument.

Construct	Items
Emerging Treatments related Internet Health Information Seeking	1. I frequently seek emerging treatments related Internet health information to understand a health problem or an illness. 2. I frequently seek emerging treatments related Internet health information to obtain different points of view from those offered by mainstream medicine. 3. I frequently seek emerging treatments related Internet health information to prevent illness by adopting a healthy lifestyle. 4. I frequently seek emerging treatments related Internet health information to find a specific solution to or treatment for a health problem. 5. I frequently seek emerging treatments related Internet health information to help a friend or family member who is ill.
Mature Treatments related Internet Health Information Seeking	1. I frequently seek mature treatments related Internet health information to understand a health problem or an illness. 2. I frequently seek mature treatments related Internet health information to obtain different points of view from those offered by mainstream medicine. 3. I frequently seek mature treatments related Internet health information to prevent illness by adopting a healthy lifestyle. 4. I frequently seek mature treatments related Internet health information to find a specific solution to or treatment for a health problem. 5. I frequently seek mature treatments related Internet health information to help a friend or family member who is ill.
Perceived Quality of Internet Health Information	**Relevance** 1. For your health information needs, to what degree do you believe the Internet Health Information (IHI) provided by the website was applicable to your needs? 2. For your health information needs, to what degree do you believe Internet Health Information (IHI) provided by the website was related to your needs? 3. For your health information needs, to what degree do you believe Internet Health Information (IHI) provided by the website was pertinent to your needs? 4. For your health information needs, to what degree do you believe Internet Health Information (IHI) provided by the website was relevant to your needs? **Understandability** 1. For your health information needs, to what degree do you believe Internet Health Information (IHI) provided by the website was clear in meaning? 2. For your health information needs, to what degree do you believe Internet Health Information (IHI) provided by the website was easy to read? 3. For your health information needs, to what degree do you believe Internet Health Information (IHI) provided by the website was easy to comprehend? 4. For your health information needs, to what degree do you believe Internet Health Information (IHI) provided by the website was understandable? **Adequacy** 1. For your health information needs, to what degree do you believe Internet Health Information (IHI) provided by the website was sufficient? 2. For your health information needs, to what degree do you believe Internet Health Information (IHI) provided by the website was complete? 3. For your health information needs, to what degree do you believe Internet Health Information (IHI) provided by the website was adequate? 4. For your health information needs, to what degree do you believe Internet Health Information (IHI) provided by the website contained the necessary topics/categories? **Usefulness** 1. For your health information needs, to what degree do you believe Internet Health Information (IHI) provided by the website was informative? 2. For your health information needs, to what degree do you believe Internet Health Information (IHI) provided by the website was valuable? 3. For your health information needs, to what degree do you believe Internet Health Information (IHI) provided by the website was helpful? 4. For your health information needs, to what degree do you believe Internet Health Information (IHI) provided by the website was useful?
Satisfaction with Internet Health Information	1. The information available on the Internet is reliable. 2. The information available on the Internet is complete. 3. The information available on the Internet is clear and understandable. 4. The information available on the Internet is well structured. 5. This doctor was interested in me as a person and not just my illness. 6. It is quite simple to surf the Internet. 7. I quickly became comfortable surfing the Internet. 8. I can easily find the information I need on the Internet. 9. The Internet is a useful tool. 10. The Internet satisfies all of my health information needs. 11. The Internet always has an answer to my question.
Psychological Safety	1. In treatment, I am still not afraid to be myself. 2. I am afraid to express my opinions in treatment. 3. I think there is a threatening medical environment.
Trust in Physician	1. In general, doctors care about their patient’s health just as much as, or more than their patients do. 2. Sometimes doctors care more about what is convenient for them than about their patient’s medical needs. 3. Doctors are extremely thorough and careful 4. I completely trust doctor’s decisions about which medical treatments are best. 5. Doctors are totally honest in telling their patients about all of the treatment options available for their conditions. 6. Sometimes doctors do not pay full attention to what patients are trying to tell them. 7. Doctors always use their very best skill and effort on behalf of their patients. 8. I have no worries about putting my life in hands of doctors. 9. A doctor would never mislead you about anything. 10. All in all, I trust doctors completely.

## References

[1] Blomstedt P., Sjöberg R.L., Hansson M., Bodlund O., Hariz M.I. (2015). Deep brain stimulation in the treatment of depression. Acta Psychiatr. Scand..

[2] Choi H.H., Cho Y.-S. (2016). Fecal microbiota transplantation: Current applications, effectiveness, and future perspectives. Clin. Endosc..

[3] Song H., Lahood N., Mostaghimi A. (2017). Intravenous immunoglobulin as adjunct therapy for refractory pyoderma gangrenosum: Systematic review of cases and case series. Br. J. Dermatol..

[4] Bylund C.L., Gueguen J.A., Sabee C.M., Imes R.S., Li Y., Sanford A.A. (2007). Provider-patient dialogue about Internet health information: An exploration of strategies to improve the provider-patient relationship. Patient Educ. Couns..

[5] Bell R.A., Hu X., Orrange S.E., Kravitz R.L. (2011). Lingering questions and doubts: Online information-seeking of support forum members following their medical visits. Patient Educ. Couns..

[6] Harris P.R., Sillence E., Briggs P. (2011). Perceived threat and corroboration: Key factors that improve a predictive model of trust in Internet-based health information and advice. J. Med. Internet Res..

[7] Seçkin G., Yeatts D., Hughes S., Hudson C., Bell V. (2016). Being an informed consumer of health information and assessment of electronic health literacy in a national sample of Internet users: Validity and reliability of the e-HLS instrument. J. Med. Internet Res..

[8] Graffigna G., Barello S., Bonanomi A., Riva G. (2017). Factors affecting patients’ online health information—Seeking behaviours: The role of the Patient Health Engagement (PHE) Model. Patient Educ. Couns..

[9] Marcinkiewicz M., Mahboobi H. (2009). The impact of the Internet on the doctor-patient relationship. Australas. Med. J..

[10] Mah J.K. (2016). Current and emerging treatment strategies for Duchenne muscular dystrophy. Neuropsychiatr. Dis. Treat..

[11] Dean C.A., Geneus C.J., Rice S., Johns M., Quasie-Woode D., Broom K., Elder K. (2017). Assessing the significance of health information seeking in chronic condition management. Patient Educ. Couns..

[12] Laugesen J., Hassanein K., Yuan Y.F. (2015). The impact of Internet health information on patient compliance: A research model and an empirical study. J. Med. Internet Res..

[13] Jiang S. (2018). How does patient-centered communication improve emotional health? An exploratory study in China. Asian J. Commun..

[14] Samal L., Saha S., Chander G., Korthuis P.T., Sharma R.K., Sharp V., Cohn J., Moore R.D., Beach M.C. (2011). Internet health information seeking behavior and antiretroviral adherence in persons living with HIV/AIDS. AIDS Patient Care STDs.

[15] Tustin N. (2010). The role of patient satisfaction in online health information seeking. J. Health Commun..

[16] Lu X., Zhang R., Wu W., Shang X., Liu M. (2018). The relationship between Internet health information and patient compliance based on trust: An empirical study. J. Med. Internet Res..

[17] Krot K. Online Information Seeking and Trust in Doctors: An Empirical Study. Proceedings of the 24th International Scientific Conference on Economic and Social Development—Managerial Issues in Modern Business.

[18] Maloney E.K., D’Agostino T.A., Heerdt A., Dickler M., Li Y., Ostroff J.S., Bylund C.L. (2015). Sources and types of online information that breast cancer patients read and discuss with their doctors. Palliat. Support. Care.

[19] Wilson T.D. (2000). Human information behavior. Inform. Sci..

[20] Dobransky K., Hargittai E. (2012). Inquiring minds acquiring wellness: Uses of online and offline sources for health information. Health Commun..

[21] Neter E., Brainin E. (2012). eHealth literacy: Extending the digital divide to the realm of health information. J. Med. Internet Res..

[22] Suri V.R., Majid S., Chang Y.K., Foo S. (2016). Assessing the influence of health literacy on health information behaviors: A multi-domain skills-based approach. Patient Educ. Couns..

[23] Hodgetts D., Bolam B., Stephens C. (2005). Mediation and the construction of contemporary understandings of health and lifestyle. J. Health Psychol..

[24] Li F., Li M., Guan P., Ma S., Cui L. (2015). Mapping publication trends and identifying hot spots of research on Internet health information seeking behavior: A quantitative and co-word biclustering analysis. J. Med. Internet Res..

[25] Yang H., Guo X., Wu T. (2015). Exploring the influence of the online physician service delivery process on patient satisfaction. Decis. Support Syst..

[26] Nath C., Huh J., Adupa A.K., Jonnalagadda S.R. (2016). Website sharing in online health communities: A descriptive analysis. J. Med. Internet Res..

[27] Richardson A., Allen J.A., Xiao H., Vallone D. (2012). Effects of race/ethnicity and socioeconomic status on health information-seeking, confidence, and trust. J. Health Care Poor Underserv..

[28] Jiang S., Street R.L. (2017). Pathway linking Internet health information seeking to better health: A moderated mediation study. Health Commun..

[29] Berger M., Wagner T.H., Baker L.C. (2005). Internet use and stigmatized illness. Soc. Sci. Med..

[30] Pang P.C., Chang S., Verspoor K., Pearce J. (2016). Designing health websites based on users’ web-based information–seeking behaviors: A mixed-method observational study. J. Med. Internet Res..

[31] Salancik G.R., Pfeffer J.A. (1978). Social information processing approach to job attitudes and task design. Adm. Sci. Q..

[32] Young G.J., Meterko M.M., Mohr D., Shwartz M., Lin H. (2009). Congruence in the assessment of service quality between employees and customers: A study of a public health care delivery system. J. Bus. Res..

[33] Hsiung H.H., Tsai W.C. (2017). The joint moderating effects of activated negative moods and group voice climate on the relationship between power distance orientation and employee voice behavior. Appl. Psychol..

[34] Higgins E.T. (1997). Beyond pleasure and pain. Am. Psychol..

[35] Liu J., Modrek S., Anyanti J., Nwokolo E., Cruz A.D.L., Schatzkin E., Isiguzo C., Ujuju C., Montagu D. (2014). How do risk preferences relate to malaria care-seeking behavior and the acceptability of a new health technology in Nigeria. BMC Health Serv. Res..

[36] Edmondson A. (1999). Psychological safety and learning behavior in work teams. Adm. Sci. Q..

[37] Edmondson A.C., Lei Z. (2014). Psychological safety: The history, renaissance, and future of an interpersonal construct. Annu. Rev. Organ. Psychol. Organ. Behav..

[38] Halbesleben J.R., Leroy H., Dierynck B., Simons T., Savage G.T., McCaughey D., Leon M.R. (2013). Living up to safety values in health care: The effect of leader behavioral integrity on occupational safety. J. Occup. Health Psychol..

[39] Collins C.J., Smith K.G. (2006). Knowledge exchange and combination: The role of human resource practices in the performance of high-technology firms. Acad. Manag. J..

[40] Triplett S.M., Loh J.M.I. (2017). The moderating role of trust in the relationship between work locus of control and psychological safety in organisational work teams. Aust. J. Psychol..

[41] Yanchus N.J., Derickson R., Moore S.C., Bologna D., Osatuke K. (2014). Communication and psychological safety in veterans health administration work environments. J. Health Organ. Manag..

[42] Wang Y.Y., Liu W., Shi H., Liu C., Wang Y. (2017). Measuring patient safety culture in maternal and child health institutions in China: A qualitative study. BMJ Open.

[43] Ducket J., Hunt K., Munro N., Sutton M. (2016). Does distrust in providers affect health–care utilization in China?. Health Policy Plan..

[44] Erden Z., Krogh G.V., Kim S. (2012). Knowledge sharing in an online community of volunteers: The role of community munificence. Eur. Manag. Rev..

[45] Lee Y.Y., Lin J.L. (2009). The effects of trust in physician on self-efficacy, adherence and diabetes outcomes. Soc. Sci. Med..

[46] Lee Y.Y., Lin J.L. (2009). Trust but verify: The interactive effects of trust and autonomy preferences on health outcomes. Health Care Anal..

[47] Zhao D.H., Rao K.Q., Zhang Z.R. (2016). Patient trust in physicians: Empirical evidence from Shanghai, China. Chin. Med. J..

[48] Chunli S., Torsten B., Sven V. (2012). Antecedents of employee’s preference for knowledge-sharing tool. Int. J. Hum. Resour. Man..

[49] Mcallister D.J. (1995). Affect- and cognition-based trust as foundations for interpersonal cooperation in organizations. Acad. Manag. J..

[50] Rodríguez V., Andrade A.D., Garcíaretamero R., Anam R., Rodríguez R., Lisigurski M., Sharit J., Ruiz J.G. (2013). Health literacy, numeracy, and graphical literacy among veterans in primary care and their effect on shared decision making and trust in physicians. J. Health Commun..

[51] Thom D.H., Hall M.A., Pawlson L.G. (2004). Measuring patients’ trust in physicians when assessing quality of care. Health Aff..

[52] Graham J.L., Shahani L., Grimes R.M., Hartman C., Giordano T.P. (2015). The influence of trust in physicians and trust in the healthcare system on linkage, retention, and adherence to HIV care. AIDS Patient Care STDs.

[53] Doescher M.P., Saver B.G., Franks P., Fiscella K. (2000). Racial and ethnic disparities in perceptions of physician style and trust. Arch. Fam. Med..

[54] Jones D.E., Carson K.A., Bleich S.N., Cooper L.A. (2012). Patient trust in physicians and adoption of lifestyle behaviors to control high blood pressure. Patient Educ. Couns..

[55] Rawaf M.M., Kressin N.R. (2007). Exploring racial and sociodemographic trends in physician behavior, physician trust and their association with blood pressure control. J. Natl. Med. Assoc..

[56] Ha H.Y., John J., John J.D., Chung Y.K. (2016). Temporal effects of information from social networks on online behavior. Internet Res..

[57] Lapidot Y., Kark R., Shamir B. (2007). The impact of situational vulnerability on the development and erosion of followers’ trust in their leader. Leadersh. Q..

[58] Blau P.M. (1964). Exchange and Power in Social Life.

[59] Foa E.B., Foa U.G., Thibault J.W., Spence J.B., Carson R.C. (1976). Resource theory of social exchange. Contemporary Topics in Sociology.

[60] Foa U.G., Foa E.B. (1974). Societal Structures of the Mind.

[61] Zhang Y., Fang Y., Wei K., Wang Z. (2012). Promoting the intention of students to continue their participation in e-learning systems. Inf. Technol. People.

[62] Gordon H.S., Pugach O., Berbaum M.L., Ford M.E. (2014). Examining patients’ trust in physicians and the VA healthcare system in a prospective cohort followed for six-months after an exacerbation of heart failure. Patient Educ. Couns..

[63] Lemire M., Paré G., Sicotte C., Harvey C. (2008). Determinants of Internet use as a preferred source of information on personal health. Int. J. Med. Inform..

[64] Chikoko G.L., Buitendach J.H., Kanengoni H. (2014). The psychological conditions that predict work engagement among tertiary education employees. J. Psychol. Afr..

[65] Torbit L.A., Albiani J.J., Aronson M., Holter S., Semotiuk K., Cohen Z., Hart T.L. (2016). Physician trust moderates the relationship between intolerance of uncertainty and cancer worry interference among women with Lynch syndrome. J. Behav. Med..

[66] Sardeshmukh S., Vandenberg R.J. (2017). Integrating moderation and mediation: A structural equation modeling approach. Organ. Res. Methods.

[67] Bu X.Q., You L.M., Li Y., Liu K., Zheng J., Yan T.B., Chen S.X., Zhang L.F. (2016). Psychometric properties of the Kessler 10 scale in Chinese parents of children with cancer. Cancer Nurs..

[68] Xiao Y.Y., Li T., Xiao L., Wang S.W., Wang S.Q., Wang H.X., Wang B.B., Gao Y.L. (2017). The Chinese version of instrument of professional attitude for student nurses (IPASN): Assessment of reliability and validity. Nurse Educ. Today.

[69] Wong W.S., Chen P.P., Chow Y.F., Wong S., Fielding R. (2016). A study of the reliability and concurrent validity of the Chinese version of the pain medication attitude questionnaire (ChPMAQ) in a sample of Chinese patients with chronic pain. Pain Med..

[70] Wimble M. (2016). Understanding health and health–related behavior of users of Internet health information. Telemed. J. E-Health.

[71] Buysse H.E., Coorevits P., Van Maele G., Hutse A., Kaufman J., Ruige J., De Moor G.J. (2010). Introducing telemonitoring for diabetic patients: Development of a telemonitoring ‘Health Effect and Readiness’ questionnaire. Int. J. Med. Inform..

[72] Biasutti M., Frate S. (2016). A validity and reliability study of the attitudes toward sustainable development scale. Environ. Educ. Res..

[73] Erdogan M., Ok A., Marcinkowski T.J. (2012). Development and validation of children’s responsible environmental behavior scale. Environ. Educ. Res..

[74] Kaiser H.F. (1970). A second generation little jiffy. Psychometrika.

[75] Kurtuldu M.K., Bulut D. (2017). Development of a self–efficacy scale toward piano lessons. Educ. Sci. Theory Pract..

[76] Bentler P.M., Bonett D.G. (1980). Significance tests and goodness of fit in the analysis of covariance structures. Psychol. Bull..

[77] Bentler P.M., Kano Y. (1990). On the equivalence of factors and components. Multivar. Behav. Res..

[78] Mu G.M., Hu Y. (2016). Validation of the Chinese version of the 12-item child and youth resilience measure. Child. Youth Serv. Rev..

[79] Polanco N.T., Solinis R.N., Arce R.S., Zabalegui I.B. (2012). Development of a questionnaire to assess the collaboration between clinicians from different levels of care. Int. J. Integr. Care.

[80] Steiger J.H., Lind J. Statistically based tests for the number of common factors. Proceedings of the Annual Meeting of the Psychometric Society.

[81] Fornell C., Larcker D.F. (1981). Evaluating structural equation models with unobservable variables and measurement error. J. Mark. Res..

[82] Sobral M.P., Costa M.E., Schmidt L., Martins M.V. (2016). COMPI Fertility Problem Stress Scales is a brief, valid and reliable tool for assessing stress in patients seeking treatment. Hum. Reprod..

[83] Jiang S., Street R.L. (2017). Factors influencing communication with doctors via the Internet: A cross–sectional analysis of 2014 HINTS survey. Health Commun..

[84] Kowalski C., Nitzsche A., Scheibler F., Steffen P., Albert U.S., Pfaff H. (2009). Breast cancer patients’ trust in physicians: The impact of patients’ perception of physicians’ communication behaviors and hospital organizational climate. Patient Educ. Couns..

